# Distinct Role of Dural and Leptomeningeal Macrophages in Maintaining Cerebrospinal Fluid Drainage to Meningeal Lymphatic Vessels

**DOI:** 10.1016/j.ajpath.2025.05.017

**Published:** 2025-06-18

**Authors:** Vikrim Lohat, Raffay Ilyas, Qing Wei, Darellynn Oo, Jingna Xue, Isabelle Horsman, Keith Keane, Matthew Stephens, Pierre-Yves von der Weid, Shan Liao

**Affiliations:** ∗ Department of Microbiology, Immunology and Infectious Diseases, Snyder Institute for Chronic Diseases, Cumming School of Medicine, University of Calgary, Calgary, Alberta, Canada; † Department of Physiology and Pharmacology, Snyder Institute for Chronic Diseases, Cumming School of Medicine, University of Calgary, Calgary, Alberta, Canada

## Abstract

Cerebrospinal fluid (CSF) drains along the perivascular space, known as the glymphatic system, to the meninges, where meningeal lymphatic vessels (MLVs) remove toxic products with excess CSF from the brain. Macrophages are widely present in the leptomeninges and dura mater of meninges. However, whether leptomeningeal and dural macrophages play the same or distinct roles in maintaining optimal CSF drainage remains unclear. Intracisterna magna injection of clodronate liposomes indicated a comprehensive depletion of leptomeningeal macrophages, a selective reduction in dural sinus-associated macrophages, a decreased density of MLVs, and disrupted CSF drainage. Macrophage depletion was associated with the infiltration of monocytes and the recovery of monocyte-derived macrophages. By day 14 after clodronate liposome, although both dural macrophages and MLVs had recovered, leptomeningeal macrophages and CSF drainage had not been restored. Furthermore, i.p. injection of anti–colony-stimulating factor 1 receptor antibody selectively depleted macrophages in the dura mater but not in the leptomeninges, without affecting MLVs or CSF drainage. The study suggests that leptomeningeal macrophages, distinct from the dural macrophages, are essential for CSF drainage to the MLVs.

## Graphical abstract

**Figure:**
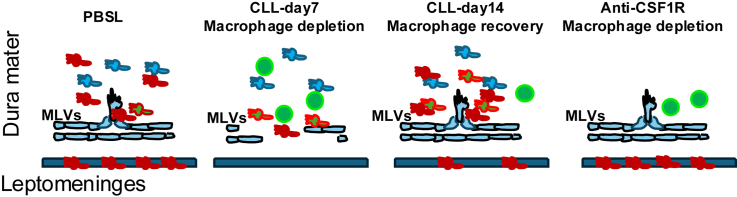

In the brain, the glymphatic system facilitates the clearance of extra cerebrospinal fluid (CSF) and brain interstitial fluid to the meninges. The meningeal lymphatic vessels (MLVs), located in the dura mater of the meninges, act as a route to drain excess CSF and interstitial fluid and associated toxic compounds from the central nervous system (CNS) to the cervical lymph nodes.^1^, ^2^, ^3^, ^4^ As such, dysfunction of MLVs and glymphatic system has been directly linked to diminished clearance of toxic brain solutes, contributing to numerous age-associated CNS disorders, such as Parkinson disease and Alzheimer disease.^4^, ^5^, ^6^ For instance, the ablation of MLVs by photoconversion of verteporfin (Visudyne) in a mouse Alzheimer disease model recapitulates the dural tissue amyloid-β deposition pathologies observed in patients with Alzheimer disease.^4^ Diminished brain fluid clearance from a similar MLV blockage in a Parkinson disease–like mouse model contributes to a more aggravated disease profile with an increased accumulation of α-synuclein.^6^ However, abolishing MLVs with adeno-associated virus–delivered soluble vascular endothelial growth factor (VEGF) receptor 3 does not show the increase in amyloid-β deposition,^7^ adding complexity to the role of MLVs in handling toxic proteins. Thus, despite the pivotal role of CSF drainage in ensuring CNS health, more studies are needed to elucidate the mechanism by which optimal CSF drainage is maintained in adult mice, which will help develop novel therapeutic targets to improve toxic protein clearance in diseases.^1^, ^2^, ^3^, ^4^^,^^8^

In contrast to peripheral lymphatic vessels, dorsal MLVs are sensitive to disruptions in VEGF-C and VEGF receptor 3 signaling, suggesting that they require continuous VEGF-C signaling to maintain their structure.^9^^,^^10^ Notably, along the dorsal MLVs, several regions have more effective fluid uptake, referred to as hotspot areas.^10^ Hotspots are areas of high sensitivity to environmental change, where lymphangiogenesis preferentially occurs during traumatic brain injury and aging.^11^^,^^12^ Given MLVs' heightened sensitivity to environmental change, the cell types needed to modulate and support the environment surrounding MLVs remain to be clarified.

Tissue-resident macrophages, tasked with prototypical roles, such as immune surveillance, antigen presentation, and removal of toxic debris, are key players in tissue homeostasis.^13^, ^14^, ^15^, ^16^ Investigation of their homeostatic role has classified macrophages as extracellular matrix (ECM) remodelers, with evidence showing that macrophages can produce proteins, such as matrix metalloproteinases (MMPs), to regulate collagen and ECM turnover.^13^, ^14^, ^15^ This unique regulatory ability of macrophages supports vasculature, as the maintenance of the ECM can allow for adequate arterial function.^15^ Intriguingly, Drieu et al^17^ found that brain border macrophages (perivascular and leptomeningeal macrophages) can also assist in the movement of CSF through perivascular spaces in the glymphatic system via the regulation of ECM components. Additionally, macrophages have a role in collagen turnover in the dura mater, as dural macrophages in adult rats localize near collagen IV fibers and internalize collagen.^18^ Presented with CNS macrophages capable of regulating tissue homeostasis through ECM remodeling, a more in-depth examination of dural and leptomeningeal macrophages is warranted when assessing a population of interest that supports CSF drainage to MLVs.

Beyond the phenotypic heterogeneity in the roles that tissue-resident macrophages play during homeostasis, it is also important to consider the changing landscape of dural macrophage populations from birth to adulthood. CNS is initially seeded by a common embryonic yolk sac progenitor, which gives rise to self-replenishing microglia and meningeal macrophage populations.^16^ Gradually, as life progresses, hematopoietic bone marrow–derived cells replace these embryonic dural macrophages.^19^ The bone marrow also provides the bone marrow–derived cells that seed dural macrophages in adult mice.^20^ Despite the predominant population of adult dural macrophages originating from bone marrow–derived cells, embryonic dural macrophages persist, allowing for a heightened self-renewal capacity for macrophages in the dura mater.^19^ Also, with the transition from postnatal to adult mice, the dural macrophage populations change morphology from a round ameboid to an elongated shape that prefers to reside along blood vasculature.^21^ The changing morphology during mice development could be attributed to a new population of macrophages emerging as major histocompatibility complex (MHC) II^+^ dural macrophages that potentially differentiate from bone marrow–derived cells and thereby diluting the neonatal MHCII^−^ dural macrophage population.^22^ Using this MHCII subdivision, Mrdjen et al^23^ have further discriminated adult macrophage populations with the expression of a lymphatic vessel marker, lymphatic vessel endothelial hyaluronan receptor 1 (LYVE-1), to subdivide dural macrophages into LYVE-1^−^MHCII^+^ or LYVE-1^+^MHCII^−^ populations. With the discrimination of multiple subtypes of dural macrophages present from birth to adulthood, further investigation is required to narrow down which, if any, macrophage population can maintain MLVs or regulate CSF drainage to MLVs.

Intracisterna magna (i.c.m.) injections of clodronate liposomes (CLLs) depleted both leptomeningeal and dura sinus–associated macrophages and disrupted MLVs and the drainage of CSF. In contrast, i.p. injections of anti–colony-stimulating factor 1 receptor (aCSF1R) antibody selectively depleted dural macrophages without affecting MLVs or CSF drainage. Thus, the study showed the distinct role of leptomeningeal and dural macrophages in maintaining optimal CSF drainage.

## Materials and Methods

### Animals

C57BL/6 mice were purchased from the Jackson Laboratory (Bar Harbor, ME). All experiments conducted for this study used 8- to 12-week–old male mice. All animal protocols were reviewed and approved by the University of Calgary (Calgary, AB, Canada) Animal Care and Ethics Committee and conformed to the guidelines established by the Canadian Council on Animal Care.

### Clodronate Liposome Treatment

Male C57BL/6 mice were anesthetized with a ketamine/xylazine mix before the procedure. Via i.c.m. administration, either CLL or control phosphate-buffered saline (PBS) liposome (PBSL), purchased from Liposoma Research (CP-005-005; Amsterdam, the Netherlands), was given to a mouse with a total volume of 25 μL at a rate of 5 μL per minute with a syringe pump (KDS Model 200 Series; Harvard Apparatus, Holliston, MA).

### aCSF1R Antibody Treatment

C57BL/6 mice (8 to 10 weeks old) were intraperitoneally injected with IgG control or aCSF1R antibody (50 mg/kg body weight; Biolegend, San Diego, CA) on day 0 and day 2, and the samples were collected 7 days later (day 9 after first injection).

### Immunofluorescence

#### OCT-Embedded Immunofluorescence

After collection, the brain samples were fixed in 4% paraformaldehyde in PBS overnight. Samples were then replaced with 30% sucrose and left for 24 hours. Next, samples were flash frozen in O.C.T with 100% ethanol and dry ice. Sections (20 to 30 μm thick) were obtained with a cryostat set at –30°C. Last, brain sections were fixed in acetone (–20°C) for 20 minutes and then left to air dry. Brain sections were stored at –80°C until use.

#### Whole-Mount Immunofluorescence

For whole-mount meningeal staining, the brain and skull cap were first carefully separated, leaving the dural membrane attached to the skull cap. With the dural membrane still attached to the skull cap, the samples were then fixed in 4% paraformaldehyde in PBS overnight and then stored in PBS. First, the samples were washed with PBS (3×) and then permeabilized and blocked with 3% bovine serum albumin with 0.1% Triton X-100 for 1 hour. Next, after three washes with 0.1% Triton X-100 in PBS, samples were incubated for overnight with primary antibodies plus 3% bovine serum albumin with 0.1% Triton X-100 in the mix. The next day, samples were washed three times with 0.1% Triton X-100 in PBS before incubation with secondary antibodies in 3% bovine serum albumin with 0.1% Triton X-100 for 2 to 4 hours. Next, the samples were washed three times with 0.1% Triton X-100 in PBS, then stained with DAPI, and then finally washed three more times with 0.1% Triton X-100 in PBS. The dural layer was then mechanically scrapped out of the skull cap and mounted for imaging with confocal microscopy.

### Intravital Time-Lapse Imaging of CSF Drainage to MLVs

Mice were anesthetized with a ketamine/xylazine mixture before the procedure. The scalp was surgically removed to expose the skull. Using a syringe pump (KDS Model 200 Series; the LabWorld Group, Hudson, MA), 5 μL of a fluorescein isothiocyanate (FITC)–dextran (10 μg/μL) and Alexa 647–ovalbumin (OVA) mixture (2 μg/μL) was i.c.m. injected at 1 μL/minute. Intravital time-lapse imaging of FITC-dextran was performed for 60 minutes using stereomicroscopy for the entire skull for all the detectable transcranial signal. At the end of the time-lapse imaging, the transcranial signals of FITC-dextran and Alexa 647–OVA were acquired as the end point signal. Mice were then euthanized to collect the skull, brain, and deep cervical lymph nodes (LNs), and the samples were fixed overnight with 4% paraformaldehyde in PBS. Whole-mount deep cervical LNs were imaged under a stereomicroscope. The skull samples were stained, as described above, and the dura mater samples were then isolated and mounted for imaging. Some brain samples were transferred to 30% sucrose before embedding in OCT and freezing on dry ice for later cryosectioning and staining. Other brain samples were stained whole mount with antibodies to image leptomeningeal macrophages.

### Quantitative RT-PCR

Before the collection of samples, mice were transcardially perfused with PBS. Dural samples were then harvested from skull caps with TRI Reagent (Sigma-Aldrich, St. Louis, MO) and snap frozen in liquid nitrogen for later use. Later, the samples in TRI Reagent were homogenized with tissue homogenizer. Next, mRNA was extracted following the addition of chloroform to the samples in TRI Reagent, and then mRNA was isolated with Qiagen RNeasy Mini Kit (number 74104) and DNase (number 5720637; Qiagen, Toronto, ON, Canada). Using the BIO-RAD iScript RT Supermix (number 1708841; Bio-Rad, Hercules, CA), 100 ng of RNA was converted to cDNA. Quantitative PCRs were performed with 5 ng of cDNA per reaction and SYBR Green Master Mix (from Applied Biosystems, Foster City, CA; number 100029284). Real-time PCRs were conducted with a QuantStudio 3 Real-Time PCR System (Thermo Fisher Scientific, Waltham, MA). Specifically, an annealing temperature of 55°C was used, and 45 cycles of amplification were performed. Refer to Table 1 for primer sequences used.

**Table 1:** Quantitative RT-PCR Primer Sequences

Gene	Forward primer sequence	Reverse primer sequence
*Col1*	5′-CACTGCCCTCCTGACGCATGG-3′	5′-CACGTCATCGCACACAGCCG-3′
*Col4*	5′-CCATCTGTGGACCATGGCTT-3′	5′-GCGAAGTTGCAGACGTTGTT-3′
*Mmp2*	5′-AACGGTCGGGAATACAGCAG-3′	5′-GTAAACAAGGCTTCATGGGGG3-′
*Mmp9*	5′-CTCAGAGATTCTCCGTGTCCTGTA-3′	5′-GACTGCCAGGAAGACACTTGGTTA-3′
*Pdgfb*	5′-GGAGTCGGCATGAATCGCT-3′	5′-CAGCCCCATCTTCATCTACGG-3′
*Vegfa*	5′-CACAGCAGATGTGAATGCAG-3′	5′-TTTACACGTCTGCGGATCTT-3′
*Vegfc*	5′-TTTGCCAATCACACTTCCTGC-3′	5′-ACACTGTGGTAATGTTGCTGG-3′
*Vegfd*	5′-CCTGGGACAGAAGACCACTC-3′	5′-TGAGATCTCCCGGACATGGT-3′

### Flow Cytometry

Before the collection of meningeal tissue, mice were transcardiacally perfused with PBS. The dura mater was then dissected from the skull cap and digested for 30 minutes at 37°C in 1 mL of complete Dulbecco’s modified Eagle’s medium (Sigma Life Science, St. Louis, MO; number D6046) with 10% fetal bovine serum containing 1:250 collagenase P (number 11213857001), 1:125 dispase II (Sigma; number D4693), and 1:100 DNase I (number 52779120). During digestion, the samples in the buffer were agitated every 10 minutes by pipetting up and down. After digestion, the cells were washed with fluorescence-activated cell sorting buffer. Next, an FcγRIII block (purified CD16/CD32 antibody) was added to the cells and incubated at room temperature for 5 minutes. The cells were then stained for the conjugated markers of interest for 30 minutes in the dark and on ice. Last, the cells were washed and then fixed with 2% paraformaldehyde in fluorescence-activated cell sorting buffer. After acquiring data with a BD FACSCanto (BD, Franklin Lakes, NJ), FlowJo 10.10.0 (BD) was used to analyze the data.

### Antibodies

The antibodies used for immunofluorescence and flow cytometry are listed in Table 2 and Table 3, respectively.

**Table 2:** Antibodies Used in IF Staining

Marker	Host	Company	Catalog no.
CCL21	Goat	R&D Systems (Minneapolis, MN)	AF457
CD206	Rat	Bio-Rad	MCA2235T
CD31 (PECAM-1)	Armenian Hamster	Sigma-Aldrich	MAB1398Z
F4/80	Rat	Biolegend	123102
LYVE-1	Rabbit	Cell Science	PA0846

CCL, chemokine (C-C motif) ligand; IF, immunofluorescence; LYVE-1, lymphatic vessel endothelial hyaluronan receptor 1; PECAM, platelet endothelial cell adhesion molecule 1.

**Table 3:** Antibodies Used in Flow Cytometry

Marker	Fluorophore	Company	Catalog no.
CD11b (clone M1/70)	APC-Cy7	BD	557657
CD11c (clone N418)	PE-Cy7	Biolegend	117318
CD19 (clone 1D3)	PE-Cy7	BD	552854
CD206	BV605	Biolegend	141721
CD3e (clone 145-2C11)	BV421	Biolegend	100341
CD4 (clone GK1.5)	PerCP	Biolegend	100432
CD45 (clone 30-F11)	PerCP	Biolegend	103130
CD45 (clone 30-F11)	APC-Cy7	BD	557659
CD8a (clone 53-6.7)	APC	Biolegend	100731
F4/80 (clone BM8)	APC	Biolegend	123116
L/D stain (fixed viability stain 510)	BV510	BD	564406
Ly-6C (clone HK1.4)	BV510	Biolegend	128033
Ly-6G (clone 1A8)	PE	Biolegend	127607

APC, allophycocyanin; PE, phycoerythrin; PerCP, peridinin chlorophyll protein.

### Statistical Analysis

Statistical tests in the GraphPad Prism software 8.1.1 (224) (GraphPad Software, Boston, MA) were used to test quantitative data. Results for respective tests were considered statistically significant at *P* < 0.05. Data were expressed as means ± SEM.

## Results

### LYVE-1^+^ Macrophages Are Associated with MLVs in Dural Sinuses

**Figure 1:**
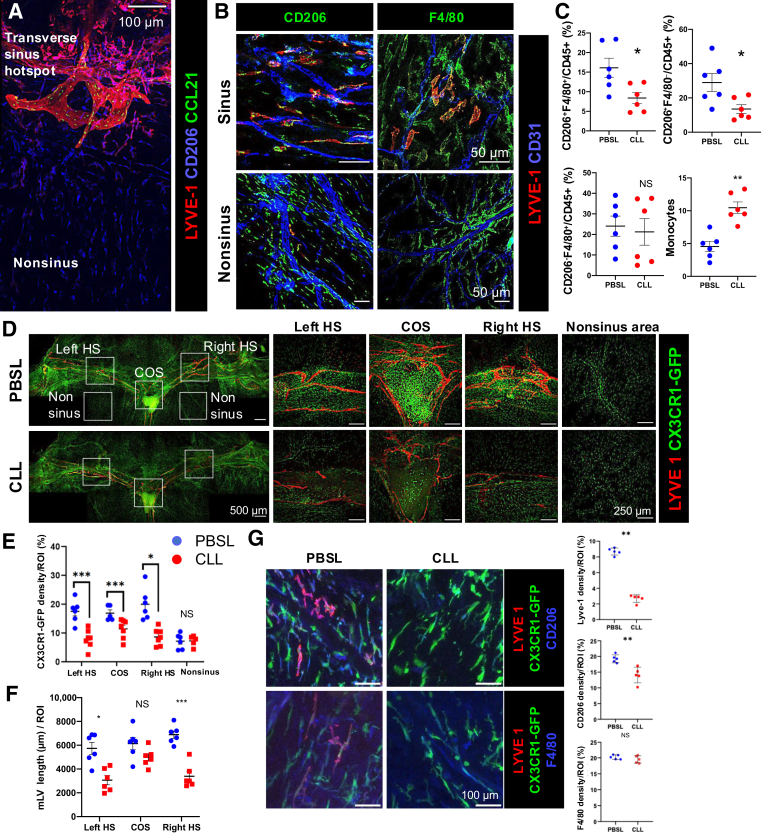
Clodronate liposome (CLL) preferentially depletes MLV-associated LYVE-1^+^ macrophages at the dural sinuses. **A:** Whole-mount staining of the dura mater shows LYVE-1^+^CD206^+^ macrophages are intimately interacting with LYVE-1^+^ chemokine (C-C motif) ligand (CCL) 21^+^ MLVs in the transverse sinus. **B:** Sinus and non–sinus-associated macrophages express different levels of LYVE-1. **C:** Three-day treatment with CLL specifically depleted macrophages as determined by flow cytometry. **D**–**F:** Whole-mount staining of the transverse sinus in transgenic *cx3cr1-gfp* reporter mice reveals a reduction in CX3CR1–green fluorescent protein (GFP)^+^ cells in the hotspots (HS) and confluence of sinus (COS) and reduced hotspot MLVs in mice 3 days after CLL treatment compared with phosphate-buffered saline liposome (PBSL) controls. **G:** Higher-magnification images showed CLL preferentially depleted LYVE-1^+^ and CD206^+^ macrophages, but less impact on F4/80+ macrophages. **C** and **G:** Two-tailed *U*-test was used. **E** and **F:** Kruskal-Wallis test was used. Data are represented as means ± SEM (**C** and **E**–**G**). ∗*P* < 0.05, ∗∗*P* < 0.01, and ∗∗∗*P* < 0.001. Scale bars: 100 μm (**A** and **G**); 50 μm (**B**); 500 μm (**D**, **left**); 250 μm (**D**, **right panels**). NS, no significance.

Macrophages in the dura mater are composed of multiple populations.^24^ Here, how different populations of macrophages are associated with MLVs was investigated. Through whole-mount immunofluorescence (IF) staining with anti–LYVE-1, anti–chemokine (C-C motif) ligand 21 (CCL21), and anti-CD206 antibodies on dural tissues collected from adult wild-type mice (8- to 12-week–old C57BL/6J mice), macrophages adjacent to MLVs were visualized (Figure 1A). Examination of macrophages distribution along MLVs in the transverse sinus (TS) and superior sagittal sinus highlighted a subpopulation of CD206^+^ CCL21^–^ macrophages (some are LYVE-1^+^ and some are LYVE-1^–^) intimately interacting with LYVE-1^+^ CD206^–^ CCL21^+^ MLVs (Figure 1A). Further confirmation with specific macrophages markers, anti-CD206 and anti-F4/80 IF staining, revealed the presence of a mixed population of LYVE-1^+^CD206^+^, LYVE-1^–^ CD206^+^, LYVE-1^+^F4/80^+^, and LYVE-1^–^F4/80^+^ macrophages in the dura sinuses (TS and superior sagittal sinus) (Figure 1B). Although both CD206^+^ and F4/80^+^ macrophages were detected in areas away from dural sinuses (nonsinus area) (Figure 1D), they were either negative for LYVE-1 or expressed it at low levels (Figure 1, A and B).

### CLL Treatment Preferentially Depletes Dural Macrophages Along Dural Sinuses

The intimate interactions between macrophages and MLVs was the impetus to assess whether macrophages play a role in maintaining MLVs in adult mice. Therefore, dural macrophages were depleted locally through the administration of CLLs via i.c.m. injection. Mice treated with PBSLs, delivered through the same route, served as a control. The CLL treatment targets macrophages and other phagocytic cells, which identify the liposome as a foreign intruder and engulfing it as a result. Once in the cell, the clodronate is released, causing the disruption of mitochondrial functioning, thereby inducing cell apoptosis.^25^ In cells isolated from the dura matter of CLL- and PBSL-treated mice investigated by flow cytometry, a decrease was observed in the proportion of two dural macrophage subsets (CD45^+^CD11b^+^CD206^+^ F4/80^+^ and CD45^+^CD11b^+^CD206^+^F4/80^–^), but not in the CD45^+^CD11b^+^CD206^–^ F4/80^+^ subset (Figure 1C). The reduction of macrophages was associated with the infiltration of monocytes (Figure 1C). Other phagocytic cells, such as neutrophils (Ly6G^+^) and dendritic cells (DCs), both the CD11c^+^CD8^+^ conventional type 1 DCs (cDC1) and CD11c^+^CD8^–^ type 2 (cDC2), were intact. At this time point, neither B cells nor T cells were affected (Supplemental Figure S1A).

Because the CLL treatment only partially depleted dural macrophages, it was critical to determine whether the CLL preferentially depleted macrophages at specific locations or distinct macrophage populations within the dura mater. Because CLL drains with the CSF to the dural sinuses, macrophages were examined in the dural sinuses 3 days after the CLL treatment by IF staining in wild-type mice. CLL treatment almost completely depleted LYVE-1^+^CD206^+^, LYVE-1^−^CD206^+^, and LYVE-1^+^F4/80^+^ macrophages within the immediate vicinity of the sinuses. However, LYVE-1^–^F4/80^+^ macrophages appeared less affected by CLL treatment (Supplemental Figure S2). To better characterize the macrophages after CLL treatment, *cx3cr1-gfp* macrophage reporter mice were used to observe the changes in macrophages in the dura mater. Consistently, at 3 days after CLL injection, the CX3CR1–green fluorescent protein (GFP)^+^ macrophages along the TS, particularly at the hotspot regions (left hotspot and right hotspot) and the confluence of sinus (COS), were preferentially reduced (Figure 1D). The macrophages away from sinuses were intact (Figure 1, D and E). At this point, the length of LYVE-1^+^ MLVs was significantly reduced in the hotspots of TS area, but not the COS in the CLL-treated mice (Figure 1F). Images with high magnification showed that LYVE-1^+^ macrophages and CD206^+^ macrophage were significantly reduced, but F4/80^+^ macrophages were less impacted (Figure 1G), consistent with the flow cytometry analysis (Figure 1C). Taken together, the results showed that the i.c.m. administration of CLL preferentially depleted dural macrophages along dural sinuses, particularly LYVE-1^+^ and CD206^+^ macrophages that reside in proximity to MLVs, while having a limited impact on macrophages in the nonsinus area. However, whether the interrupted MLVs were a result of macrophage depletion or whether CLL treatment might cause off-target damage directly to the MLVs is unclear.

### CLL Treatment Further Interrupts MLVs by 7 Days After CLL Injection

**Figure 2:**
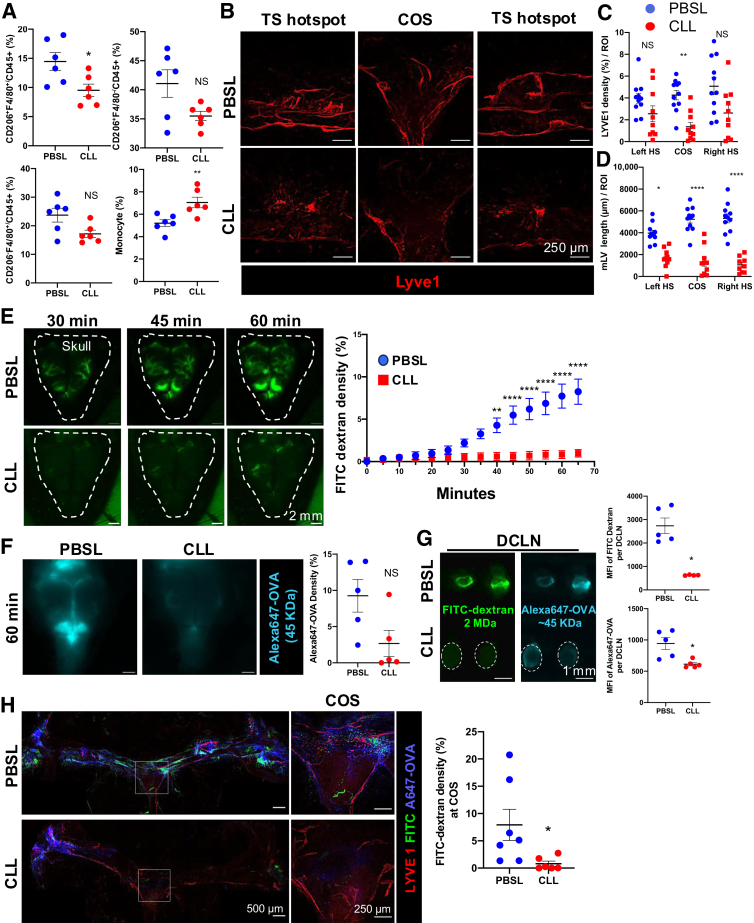
Clodronate liposome (CLL) treatment ablates MLVs and interrupts CSF drainage at 7 days after CLL injection. **A:** Macrophage and monocytes in the dura mater of day 7 CLL- and phosphate-buffered saline liposome (PBSL)–treated mice, as determined by flow cytometry. **B:** Whole-mount imaging showing anti–LYVE-1 staining at day 7 CLL-treated mice compared with PBSL controls at different regions of interest (ROIs) at the transverse sinus (TS) using *lysm-gfp* reporter mice. **C** and **D:** Quantification of LYVE-1 density (%; **C**) and total MLV length (in μm; **D**) per ROI in control and day 7 CLL-treated mice, respectively. **E**–**G:** Fluorescent tracers i.c.m. injection [Alexa647-OVA (small molecule, approximately 45 KDa) and fluorescein isothiocyanate (FITC)–dextran (macromolecule, 2 MDa)] showing CSF drainage in day 7 CLL- and PBSL-treated mice. **E:** Time-lapse imaging of skull with i.c.m. injected FITC-dextran. **Dashed lines** encircle the area of the skull to aid visualization. Two-way analysis of variance with a Sidak multiple comparison test was used. **F:** Alexa647-OVA accumulation under the skull at 60 minutes after i.c.m. injection. **G:** Accumulation of i.c.m. injected Alexa647-OVA and FITC-dextran in the deep cervical lymph nodes (LNs) of day 7 CLL- and PBSL-treated mice. **Dashed lines** encircle the area of the CLL deep cervical LNs to aid visualization. **H:** Whole-mount staining reveals a reduction in the accumulation of both i.c.m.-injected FITC-dextran and Alexa647-OVA in the transverse sinus of day 7 CLL-treated mice compared with PBSL controls. The **boxed area** on the transverse sinus represents the confluence of sinus (COS) area. **A** and **F**–**H:** Two-tailed *U*-test was used. **C** and **D:** Kruskal-Wallis test was used. Data are means ± SEM (**A** and **C**–**H**). *n* = 6 (**A**). ∗*P* < 0.05, ∗∗*P* < 0.01, and ∗∗∗∗*P* < 0.0001. Scale bars: 250 μm (**B** and **H**, **right**); 2 mm (**E** and **F**); 1 mm (**G**); 500 μm (**H, left**). MFI, mean fluorescence intensity; NS, no significance.

To further understand whether CLL treatment sustained the abrogation of macrophages and the damage to MLVs, PBSL or CLL was i.c.m. injected into wild-type mice and immune cells and MLVs were examined 7 days after the injection. The immune cell composition in the dura mater was analyzed by flow cytometry, and the results highlighted a sustained reduction of CD206^+^F4/80^+^ macrophages (Figure 2A). CD206^+^F4/80^–^ macrophage showed a trend of recovery despite monocyte proportion remaining elevated (Figure 2A). Neutrophils remained intact. However, the cDC1 population was increased, while the cDC2 population remained intact. B cells also remained intact, but more T cells, including both CD4^+^ and CD8^+^ T cells, were present in the dura mater (Supplemental Figure S1B). These results further demonstrated that i.c.m. CLL treatment did not deplete off-target phagocytic immune cells in the dura mater.

Next, using IF staining of the dura mater with anti–LYVE-1, image quantification of different regions of interest at the hotspot areas along the TS indicated that LYVE-1 density was significantly reduced in the hotspots and COS area of CLL-treated mice compared with PBSL controls (Figure 2B). However, the LYVE-1 density at each hotspot area along the TS displayed no significant difference between CLL- and PBSL-treated groups after 7 days post-CLL (Figure 2C), potentially because of the shared marker of LYVE-1 between MLVs and LYVE-1^+^ macrophages. Anti-CCL21 was used to better distinguish the LYVE-1^+^ MLVs and LYVE-1^+^ macrophages, and it was observed that some area showed LYVE-1^+^CCL21^–^ cell clusters, suggesting they were disorganized LYVE-1^+^ macrophages (Supplemental Figure S3). The pattern of LYVE-1^+^ macrophages and MLVs varied randomly in different areas at this time point (Supplemental Figure S3). To ensure that only MLVs were analyzed, the length of LYVE-1^+^ MLVs was traced in each region of interest, and the results showed a significant decrease in MLV length at COS and hotspot areas along the TS (Figure 2D). Therefore, it appeared that CLL treatment resulted in the reduction in MLVs at 7 days after CLL treatment.

### CLL Treatment Impairs CSF Drainage to MLVs and Deep Cervical Lymph Nodes

Given that i.c.m. CLL administration significantly reduced MLVs 7 days after treatment, it was next used to characterize whether the transport of CSF was correspondingly diminished. Specifically, FITC-dextran (2 MDa) and Alexa647 conjugated ovalbumin (AL647-OVA; approximately 45 KDa) were used to visualize macromolecule and small-molecule drainage, respectively. Briefly, the scalp was surgically removed and the skull was exposed, followed by the i.c.m. injections of the FITC-dextran and Alexa 647–OVA mixture (1 μL/minute; 5 μL in total injection), and intravital time-lapse imaging was performed on FITC-dextran for 60 minutes with stereomicroscopy through the exposed skull (Figure 2E, Supplemental Figure S4, and Supplemental Video S1). At the end of the time-lapse imaging, the transcranial Alexa647-OVA signal was also imaged. The draining deep cervical LNs and the dura mater were then collected for fixed sample imaging.

Time-lapse imaging showed CSF tracer (FITC-dextran) efflux from the brain toward the dura mater via arachnoid cuff exit points over time (Figure 2E and Supplemental Video S1). The transcranial distribution of FITC-dextran could be visualized at perivascular area in the brain and in the dura sinuses (Supplemental Figure S4). To better observe the distribution of FITC-dextran on different tissues, αSMA-DsRed reporter mice were used, which highlighted smooth muscle cells around the arteries and other organs. Different tissues were isolated 2 hours after i.c.m. FITC-dextran injection. FITC-dextran was observed along the perivascular space in the brain and the leptomeninges (Supplemental Figure S5, A–C). In the dura mater, FITC-dextran was concentrated in the dural sinuses (TS, COS, and superior sagittal sinus) and accumulated in the deep cervical LNs (Supplemental Figure S5, D and E).

To understand whether CLL treatment interrupts CSF drainage, the transport of FITC-dextran was compared by quantification of time-lapse imaging. At day 7, PBSL-treated mice distributed approximately eight times more FITC-dextran than the CLL-treated mice an hour after tracer administration (Figure 2E and Supplemental Videos S1 and S2). Correspondingly, AL647-OVA drainage via the glymphatic system to the dura mater varied and showed a trend of reduction but was not as substantial as FITC-dextran (Figure 2F). Images of the draining deep cervical LNs revealed a substantial reduction in FITC-dextran in the CLL-treated compared with PBSL-treated mice (Figure 2G). The reduction in Alexa647-OVA was also significant, but it appeared to be less dramatic than the FITC-dextran (Figure 2G).

Next, IF staining was performed with anti–LYVE-1 on the fixed dura mater to highlight the correlation between the tracers and the MLVs. Confocal microscopy revealed reduced accumulation of FITC-dextran and Alexa647-OVA along the diminished MLVs in the dura mater of day 7 post-CLL treated compared with the PBSL-treated mice (Figure 2H). The density of FITC-dextran tracer at the COS area was also reduced in the CLL-treated mice compared with control PBSL-treated mice (Figure 2H). Accordingly, it was confirmed that, along with the reduced macrophages and diminished MLVs, the CSF transport through the glymphatic system to MLVs and deep cervical LNs was reduced 7 days after CLL administration.

### Recovery of MLVs Is Associated with Monocyte-Derived Macrophages by 14 Days After CLL Treatment

**Figure 3:**
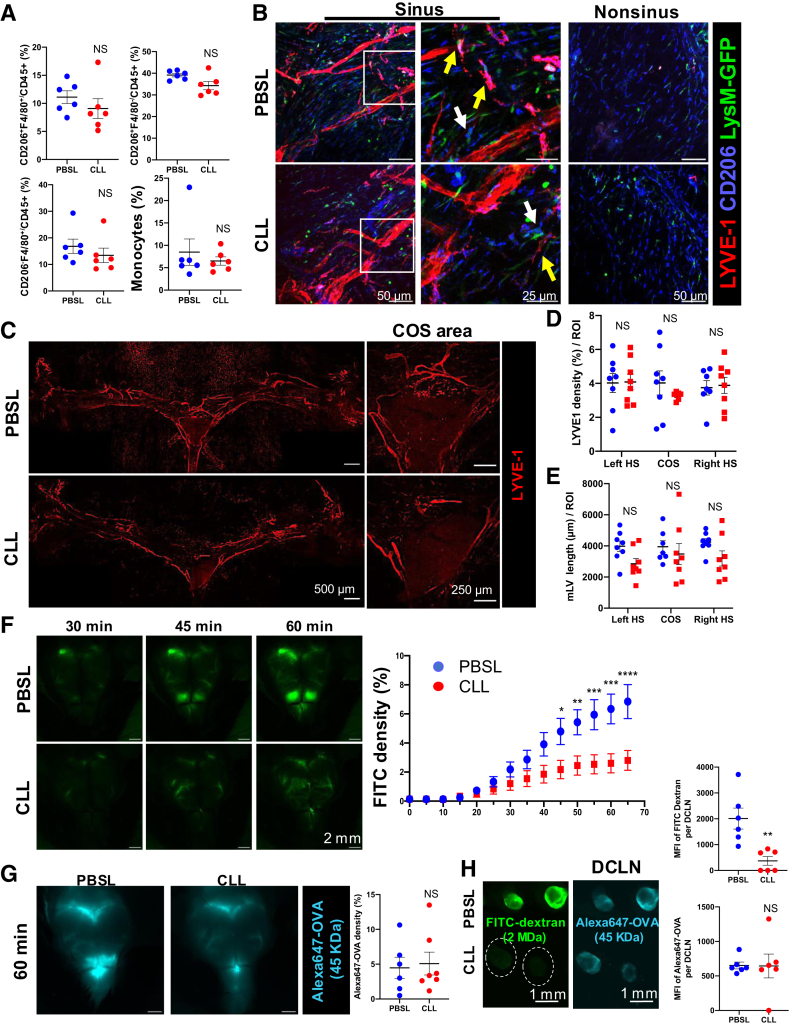
Dural macrophage and MLV recovery is not sufficient to restore CSF drainage. **A:** Macrophage and monocytes and other immune cells in the dura mater of day 14 clodronate liposome (CLL)– and phosphate-buffered saline liposome (PBSL)–treated mice as determined by flow cytometry, respectively. **B:** LysM–green fluorescent protein (GFP) reporter mice showed monocyte-derived macrophages in the dura mater. The **white boxed area** on the sinus images represents an area of interest, which was magnified in the images to the **right**. **Yellow arrows** show GFP^+^LYVE-1^+^CD206^+^ cells, and **white arrows** show GFP^+^LYVE-1^−^CD206^+^ cells. **C:** Confocal imaging showing transverse sinus LYVE-1 staining from mice 14 days after treatment with either PBSL or CLL. **D** and **E:** Quantification of LYVE-1 density (%; **D**) and total MLV length (in μm; **E**) per region of interest (ROI) in control and CLL-treated day 14 mice. **F**–**H:** Fluorescent tracers showing the accumulation of i.c.m. injected Alexa647-OVA and fluorescein isothiocyanate (FITC)–dextran in day 14 CLL- and PBSL-treated mice. **F:** Time-lapse imaging of the skull with i.c.m. injected FITC-dextran. **G:** Alexa647-OVA accumulation under the skull at 60 minutes after i.c.m. injection. (**H**) Accumulation of i.c.m. injected Alexa647-OVA and FITC-dextran in the draining cervical lymph nodes (DCLNs) of day 14 CLL- and PBSL-treated mice. **Dashed lines** encircle the area of the FITC-dextran CLL deep cervical lymph nodes to aid visualization. **A**, **G**, and **H:** Two-tailed *U*-test was used. **D** and **E:** Kruskal-Wallis test was used. **F:** Two-way ANOVA with a Sidak multiple comparison test was used. Data are means ± SEM (**A**, **D**, **E**, **G**, and **H**). ∗*P* < 0.05, ∗∗*P* < 0.01, ∗∗∗*P* < 0.001, and ∗∗∗∗*P* < 0.0001. Scale bars: 50 μm (**B**, **left and right panels**); 25 μm (**B**, **center panels**); 500 μm (**C**, **left panels**); 250 μm (**C**, **right panels**); 2 mm (**F** and **G**); 1 mm (**H**). COS, confluence of sinus; MFI, mean fluorescence intensity; NS, no significance.

As the depletion of macrophages with CLL is transient,^26^ whether macrophages and MLVs can be recovered in later stages of CLL treatment was investigated next. Because macrophage depletion is associated with monocyte infiltration and monocytes are known to be able to differentiate into macrophages in the dura mater,^20^ the macrophage and other immune cell composition were examined in the dura mater at 14 days after CLL treatment. Monocytes, macrophages, and other immune cells had regained their initial levels. However, the CD4^+^ T cells remained more abundant in the CLL- than PBSL-treated control mice at this time point (Figure 3A and Supplemental Figure S1C). Next, to verify whether monocytes can differentiate into macrophages in the dura mater after the macrophage depletion, *lysm-gfp* reporter mice were used, in which neutrophils, monocytes, and monocyte-differentiated macrophages express GFP. GFP^+^CD206^+^ macrophages were detected in the PBSL-treated mice, indicating that monocyte-macrophage differentiation contributed to macrophages in control conditions (Figure 3B). GFP^+^CD206^+^ macrophages included both LYVE1^+^ and LYVE-1^−^ macrophages, showing that monocytes could differentiate into both LYVE-1^+^ and LYVE-1^−^ macrophages in control mice (Figure 3B). Similarly, at day 14 after CLL treatment, GFP^+^ macrophages were detected near the MLVs, some of them interacting with MLVs. This observation indicates that dural macrophages could be restored by monocytes after CLL treatment (Figure 3B). GFP^+^ macrophages were also detected in the nonsinus area, indicating that monocytes also contributed to the macrophages homeostatic turnover in nonsinus area. However, GFP^+^ macrophages in the nonsinus area were either negative for LYVE-1 or expressed low levels of LYVE-1, similar to the nonsinus macrophages observed in control mice (Figure 1B and Figure 3B).

Having shown that monocytes can differentiate into dural macrophages after CLL treatment, dura maters were collected at 14 days after CLL treatment. Anti–LYVE-1 IF staining and imaging demonstrated the revival of MLVs in the dura mater at day 14 following CLL treatment (Figure 3C). Quantitative analysis substantiated the recovery of the MLVs as the investigation of LYVE-1 density and MLV length revealed no significant difference at hotspots in the TS area and COS areas between PBSL and CLL treatments (Figure 3, C–E). Therefore, the restoration of MLVs at 14 days after the administration of CLL paralleled the recovery of macrophages.

### Recovery of Dural Macrophages and MLVs Is Not Sufficient to Restore CSF Drainage to MLVs

To understand whether the restored MLVs at day 14 allowed the reestablishment of CSF drainage, intravital time-lapse imaging was performed to trace FITC-dextran and AL647-OVA after i.c.m. injection (Figure 2). FITC-dextran drainage via perivascular space to the dura mater was only partially restored (Figure 3F). Surprisingly, by the end of the intravital time-lapse imaging, AL647-OVA accumulation beneath the skull appeared to be restored (Figure 3G). Similarly, during the collection of deep cervical LNs, FITC-dextran levels were not restored, whereas AL647-OVA accumulation appeared to be recovered (Figure 3H). Thus, recovery of dural macrophage and MLV macrophages was not sufficient to restore CSF drainage. This appeared to differentially affect the drainage of FITC-dextran (2 MDa) and the AL647-OVA (approximately 45 KDa).

### Leptomeningeal Macrophage and CSF Drainage Takes Longer Time to Recover after the CLL Treatment

**Figure 4:**
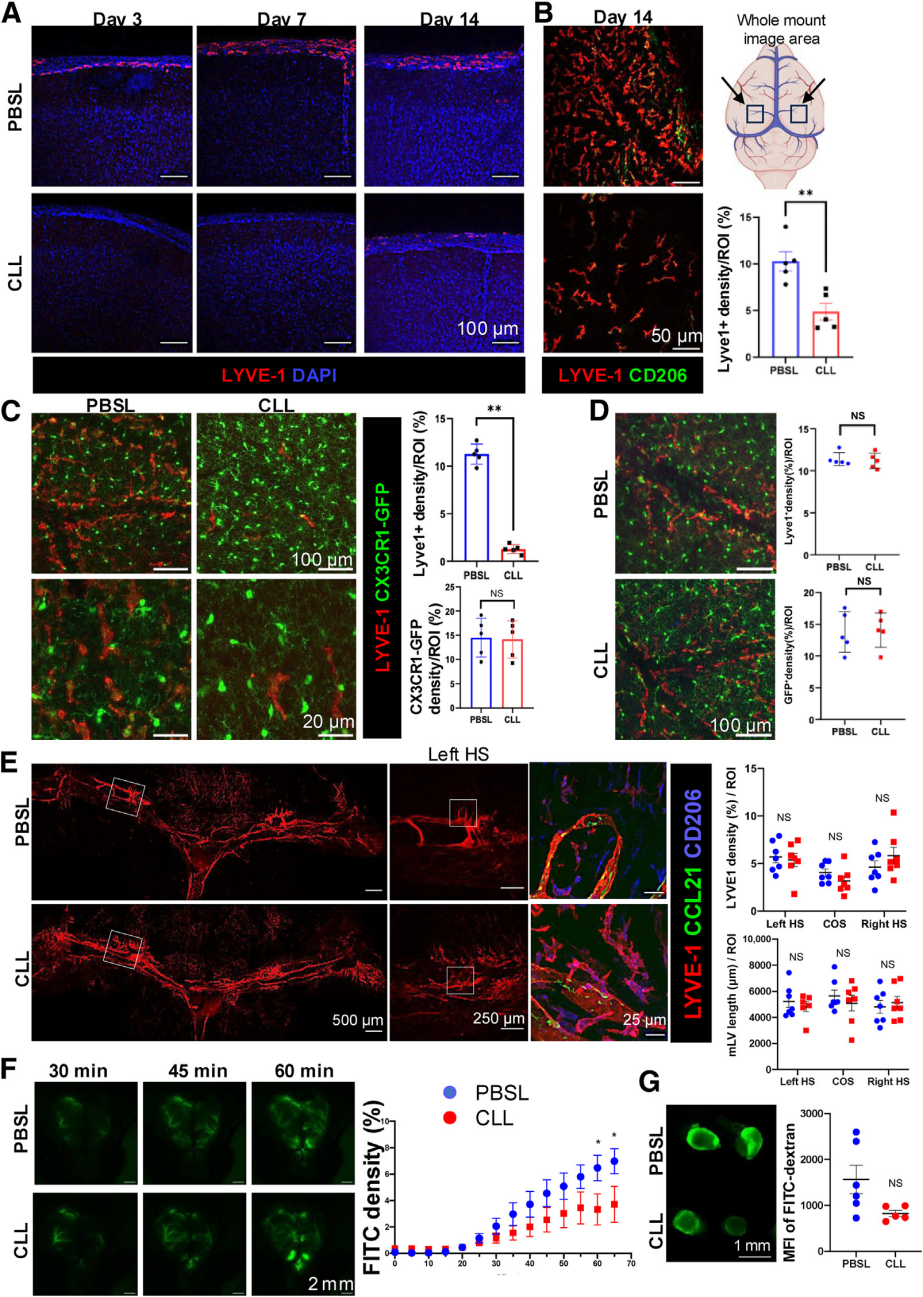
Leptomeningeal macrophages and CSF drainage take a longer time to recover after the clodronate liposome (CLL) treatment. **A** and **B:** Whole-mount staining using wild-type mice confirms that leptomeningeal macrophages were not fully restored by day 14 after CLL treatment. **B: Black arrows** on the illustration represent the area on the leptomeninges where the images were acquired. **C:***cx3cr1-gfp* reporter mice showed CLL treatment depleted leptomeningeal macrophages but not microglia at day 3. **D:***cx3cr1-gfp* reporter mice showed macrophage recovery at day 21 after CLL treatment. **E:** Day 21 after CLL treatment, dural macrophages and MLVs showed better recovery. The **boxed area** on the image of the entire transverse sinus represents the hotspot area. The **boxed area** on the left hotspot (HS) represents an area of interest, which was magnified and shown with the images to the **right**. **F:** Time-lapse imaging of exposed skull after i.c.m. fluorescein isothiocyanate (FITC)–dextran injection. **G:** Accumulation of i.c.m. injected FITC-dextran in the draining cervical lymph nodes of day 21 CLL- and phosphate-buffered saline liposome (PBSL)–treated mice. **A**–**D** and **G:** Two-tailed *U*-test was used. **D:** Kruskal-Wallis test was used. **F:** Two-way analysis of variance with a Sidak multiple comparison test was used. Data are means ± SEM (**B**–**G**). ∗*P* < 0.05, ∗∗*P* < 0.01. Scale bars: 100 μm (**A**, **C**, **top panels**, and **D**); 50 μm (**B**); 20 μm (**C**, **bottom panels**); 500 μm (**E**, **left panels**); 250 μm (**E**, **center panels**); 25 μm (**E**, **right panels**); 2 mm (**F**); 1 mm (**G**). GFP, green fluorescent protein; NS, no significance; ROI, region of interest.

CLL i.c.m. injection depletes brain border macrophages, including the perivascular and leptomeningeal macrophages, and impairs glymphatic drainage.^17^ To understand why the restoration of MLVs was not sufficient to recover CSF drainage, macrophages were visualized in the brain sections by IF staining. Leptomeningeal macrophages were depleted by i.c.m. CLL treatment as early as day 3, remained depleted at day 7, and appeared to be partially restored by day 14 after CLL injection (Figure 4A). Next, whole-mount staining and imaging of the leptomeninges (view from the top surface of the brain) were also performed. Leptomeningeal macrophages were only partially restored by day 14 after CLL injection compared with those in PBSL-treated mice (Figure 4B). To better understand the depletion and the recovery of macrophages, CX3CR1-GFP reporter mice were used. I.c.m. CLL treatment depleted leptomeningeal macrophages (LYVE-1^+^CX3CR-GFP^+^) but not microglia (LYVE-1^−^CX3CR1-GFP^+^) at day 3 after CLL injection (Figure 4C). To determine whether leptomeningeal macrophages could be restored over an extended period of time, their presence was examined 21 days after CLL injection, revealing evidence of recovery (Figure 4D). Thus, after i.c.m. CLL treatment, leptomeningeal macrophages took longer to recover than dural macrophages or MLVs.

To allow for the full recovery of CSF drainage, the examination was extended to day 21 after CLL treatment. At this later time point, the rescue of MLVs was quantitively confirmed, as the LYVE-1 density and MLV length of the day 21 group showed no significant differences between CLL and PBSL-treated mice (Figure 4E), indicating MLV restoration. Interestingly, an increased number of LYVE-1^+^ macrophages appeared to closely associate with MLVs around the restored hotspots area, similar to the pattern observed in wild-type controls (Figure 1A and Figure 4E). Next, the drainage of FITC-dextran (2 MDa) was measured on day 21 after CLL treatment. Intravital time-lapse imaging showed that FITC-dextran drainage was more effectively restored 21 days after CLL treatment, although it had not completely recovered (Figure 4F). The recovery of FITC-dextran drainage in the deep cervical LNs was consistent with the time-lapse imaging (Figure 4G). This confirmed that full restoration of CSF drainage after CLL treatment required more time than the recovery of dural macrophages and MLVs, and was associated with the recovery of leptomeningeal macrophages.

### Low-Dose CLL Treatment Shows Limited Damage to MLV or Dural Macrophages

Five μL CLL i.c.m. injection depletes brain border macrophages and impairs CSF drainage without damaging MLVs.^17^ This study used 25 μL CLL i.c.m. injection. To better understand whether high-dose CLL injection causes more damage to macrophages or to MLVs, 5 μL CLL i.c.m. was injected. Consistent with a previous report by Drieu et al*,*^17^ the 5 μL CLL treatment depleted leptomeningeal macrophages (Supplemental Figure S6), although less comprehensively than the 25 μL CLL injection (Figure 4C). The low-dose CLL treatment showed a trend of reduced MLVs, but it had limited damage to dural macrophage (Supplemental Figure S6).

### aCSF1R Antibody Treatment Selectively Depletes Dural Macrophages without Impacting MLV or CSF Drainage

**Figure 5:**
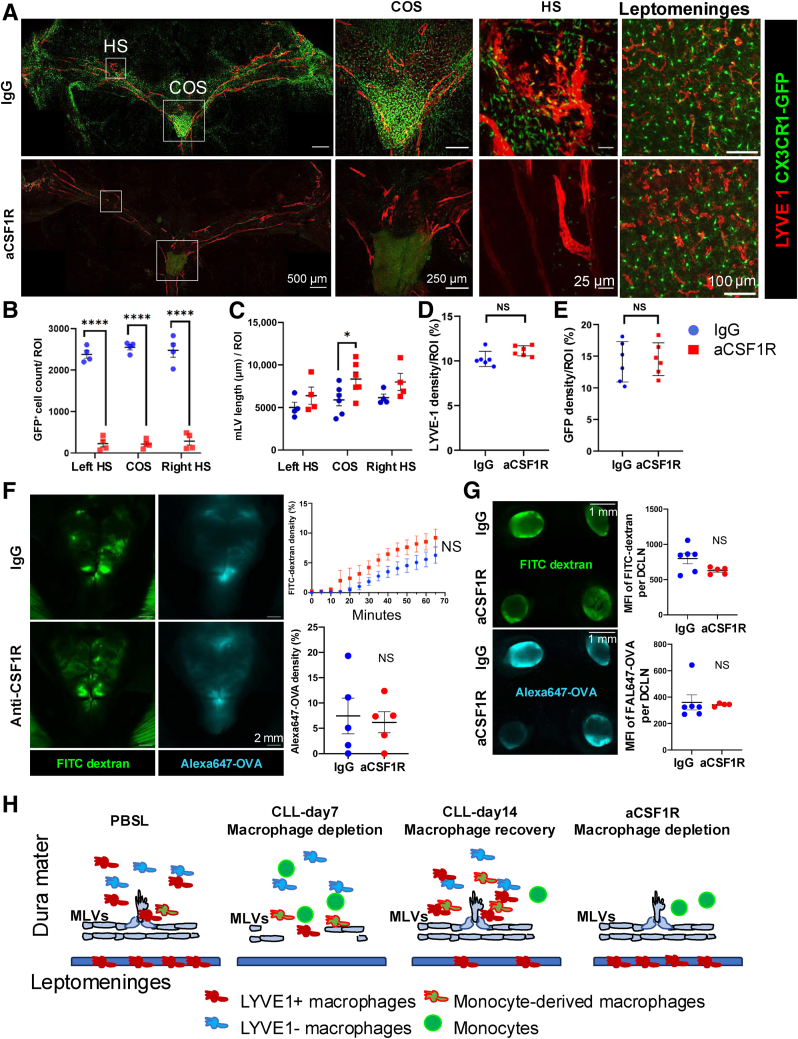
Anti–colony-stimulating factor 1 receptor (aCSF1R) i.p. injection selectively depletes dural macrophages without impacting MLVs, leptomeningeal macrophages, or CSF drainage. **A**–**E:** Using CX3CR1–green fluorescent protein (GFP) reporter mice, i.p. injection of aCSF1R comprehensively depleted dural macrophages without interruption of MLVs, leptomeningeal macrophages, or microglia. **Boxed areas** represent hotspot (HS) areas and confluence of sinus (COS) areas, as indicated on the figure. Leptomeninges scale bar = 250 μm. **B:** CX3CR1-GFP^+^ macrophages in the COS area. **C:** Length of MLVs. **D** and **E:** LYVE-1 and GFP density in the leptomeninges. **F** and **G:** Time-lapse imaging showed no difference in tracer [fluorescein isothiocyanate (FITC)–dextran and AL647-OVA] distribution between IgG or aCSF1R treated mice. **H:** Schematic model of leptomeningeal and dural macrophages in maintaining CSF drainage. In the dura mater of control mice, multiple populations of macrophages, particularly LYVE-1^+^ macrophages, are associated with MLVs. CLL treatment preferentially depleted LYVE-1^+^ macrophages in the pia mater and dura sinuses, impairing CSF drainage and MLVs by day 7 after CLL treatment. Monocytes infiltrated the dura mater, replenished the dural macrophages, and restored MLVs, but leptomeningeal macrophages take a longer time to recover, which is associated with long time to restore the CSF drainage by day 14. I.p. injection of aCSF1R antibody depleted dural macrophages without impact leptomeningeal macrophages, MLVs, or CSF drainage. **B** and **C:** Kruskal-Wallis test was used. **D**, **E**, **F** (**bottom**), and **G:** Two-tailed *U*-test was used. **F** (**top**)**:** Two-way analysis of variance with a Sidak multiple comparison test was used. Data are means ± SEM (**B**–**G**). ∗*P* < 0.05, ∗∗∗∗*P* < 0.0001. Scale bars: 500 μm (**A**, **first column**); 250 μm (**A**, **second column**); 25 μm (**A**, **third column**); 100 μm (**A**, **fourth column**); 2 mm (**F**); 1 mm (**G**). NS, no significance; ROI, region of interest.

Twenty-five μL of CLL caused more damage to dural macrophages and MLVs than 5 μL of CLL, which raised the question as to whether a high dose of CLL treatment might directly damage MLVs. Here, a noninvasive method was developed to selectively deplete dural macrophages to distinguish the roles of leptomeningeal and dural macrophages in regulating CSF drainage and MLVs. Given that the dura mater lacks a blood-brain barrier and that the blood-brain barrier may prevent antibody entry into the brain and leptomeninges, an i.p. injection of aCSF1R antibody was employed to deplete dural macrophages. IgG control and aCSF1R antibody (50 mg/kg) were injected on days 0 and 2, and brain and dura mater were collected 7 days later. In CX3CR1-GFP macrophage reporter mice, aCSF1R treatment comprehensively depleted dural macrophages (Figure 5, A and B). Surprisingly, in contrast to CLL treatment, the length of MLVs was intact (Figure 5, A and C). On the other hand, in the leptomeninges, neither LYVE-1^+^CX3CR^–^GFP^+^ macrophages nor CX3CR1^–^GFP^+^ microglia were affected by the treatment (Figure 5, A, D, and E). Because MLV-associated macrophages were ablated, higher-magnification images at the hotspot region showed a relatively smooth surface of MLVs, and LYVE-1 expression on MLVs appeared dimmer in some areas (Figure 5A). To determine whether the selective depletion of dural macrophages impacted drainage of CSF, the time-lapse images were used to trace the drainage of FITC-dextran and AL647-OVA, as addressed above. Neither FITC-dextran nor AL647-OVA drainage to meninges and the deep cervical LNs was interrupted by the aCSF1R treatment (Figure 5, F and G). Thus, dural macrophages were neither essential for maintaining the length of MLVs nor for guiding CSF drainage to MLVs in adult mice.

## Discussion

Macrophages are generally considered specialized cells involved in the elimination of toxic proteins, antigen processing, and immune surveillance. This study further emphasized the alternative role of macrophages in supporting CSF drainage through the glymphatic system to MLVs. Notably, 25 μL of CLL i.c.m. treatment depleted macrophages in both the leptomeninges and dura mater, which was associated with reduced MLVs and impaired CSF drainage (Figure 5H). Subsequently, the recovery of dural macrophages and MLVs, but not leptomeningeal macrophages, by day 14 after CLL treatment suggested that dural macrophages and MLVs alone are insufficient to support CSF drainage (Figure 5H). Furthermore, a low dose of CLL (5 μL) caused less damage to leptomeningeal macrophages, with minimal impact on dural macrophages or MLVs. Importantly, noninvasive i.p. aCSF1R antibody treatment led to the selective depletion of dural macrophages, without impacting leptomeningeal macrophages, MLVs, or CSF drainage. Together, these results highlight the distinct roles of leptomeningeal and dural macrophages in supporting optimal CSF drainage (Figure 5H).

Although CLL treatment was intended to deplete macrophages, it may have also affected other phagocytic cells, including monocytes, microglial cells, and tissue-resident DCs.^27^ The experimental results indicated the specificity of CLL in the depletion of dural sinus macrophage after the i.c.m. injection, as no significant changes to neutrophils and DC populations were observed. Also, monocyte population was increased after CLL treatment. Thus, i.c.m. CLL treatment did not significantly affect other phagocytic cells in the dura mater. The preferential depletion of macrophages in hotspot regions can be attributed to the mode of CLL administration via i.c.m. injection, since CLL was drained with CSF through glymphatic system and delivered to the hotspot regions.^11^ Therefore, dural macrophages close to the hotspot areas have primary contact with liposomes. Accordingly, LYVE-1^+^ dural macrophages are likely the primary targets of CLL treatment, given their close association with MLVs in the dural sinuses, and their better phagocytic capability than that of LYVE-1^–^ macrophages, as previously reported.^17^ This is demonstrated by the preferential depletion of LYVE-1^+^ macrophages in the hotspots and COS regions (Figure 1, D and E).

Because the high-dose CLL treatment (25 μL) caused more severe damage to MLVs than the low-dose treatment (5 μL), lymphatic endothelial cells, which express LYVE-1, may have phagocytosed CLL, leading to apoptosis. Therefore, CLL i.c.m. treatment alone is not sufficient to determine whether the reduction in MLVs was due to a lack of macrophages or whether CLL treatment caused off-target damage to lymphatic endothelial cells, leading to reduced MLVs. To distinguish between these possibilities, aCSF1R was injected intraperitonially, resulting in comprehensive depletion of dural macrophages. Despite this, MLV lengths remained intact, suggesting that dural macrophages are not required for the maintenance of MLV length. However, the possibility cannot be excluded that long-term depletion of dural macrophages with aCSF1R treatment may eventually disrupt MLV integrity.

These results raised more questions regarding the function of dural macrophages. As macrophages are critical producers of lymphatic growth factors, such as VEGF-C in inflammation and cancer,^28^^,^^29^ it was originally proposed that the depletion of macrophages may limit the number of growth factors needed to maintain MLVs. However, the results revealed no change in the mRNA expression of *Vegfc* and *Vegfd* between CLL treatment and PBSL-control groups (Supplemental Figure S7). Given that the primary sources of VEGFC in the dura mater are blood vessel smooth muscle cells, pineal gland, and pituitary gland,^9^ the lack of changes to mRNA transcript levels of *Vegfc/d* following CLL treatment was not unexpected.

Recent studies supported a role for LYVE-1^+^ dural macrophages in ECM remodeling through their expression of MMPs.^14^^,^^15^^,^^30^ This proposed mechanism is supported by the evidence of reduced mRNA transcript levels of *Mmp9* in CLL-treated mice (Supplemental Figure S7). Current literature has also confirmed the role of *Mmp9* expressing LYVE-1^+^ macrophages and in collagen degradation in regulating the arterial stiffness.^15^ Although there was no difference in *Mmp2* and ECM transcripts (*Col1* and *Col4*) between CLL and control treatments (Supplemental Figure S7), significant changes may occur selectively at the dural sinuses, where dural macrophage depletion predominantly takes place. Drieu et al^17^ showed that ECM remodeling plays an essential role in regulating CSF drainage at day 7 after i.c.m. CLL treatment. However, Drieu et al^17^ showed that low dose of CLL i.c.m. treatment did not impact the structure of MLVs. Low dose of CLL had minimal impact of dural macrophages or MLVs. Regardless, the reduction of dural ECM remodeler (*Mmp9*) was observed in the high dose of CLL-treated mice, where dural LYVE-1^+^ macrophages were selectively depleted, which synchronized the disrupted CSF drainage after CLL treatment.

During the recovery phase after CLL treatment, the drainage of AL647-OVA restored faster than that of 2-MDa FITC-dextran. It is suspected that macrophages may have more significant impact on draining large molecular weight materials than on draining small molecular weight materials. However, whether macrophages impact the drainage pattern for materials of different sizes or types (proteins versus dextran) remains unclear. Furthermore, in control mice, after fixation and staining, FITC-dextran was retained in the tissue and macrophages, whereas AL647-OVA was mostly retained in the leptomeningeal macrophages in the leptomeninges (Supplemental Figure S8A). This pattern was consistently observed in the dura mater (Supplemental Figure S8B). As anticipated, in the aCSF1R-treated mice, the distribution of FITC-dextran and AL647-OVA in the leptomeninges was similar to that in the control mice. However, in the dura mater, because macrophages were comprehensively depleted, most of these materials were trapped in the tissue except a few residual macrophages retaining FITC-dextran and AL647-OVA (Supplemental Figure S8). Thus, the primary role of dural macrophages is likely to phagocytose and clear toxic proteins from the CSF, a process that is compromised in neurodegenerative diseases. However, whether the retention of materials in the tissue during macrophage depletion with aCSF1R impacts neurodegenerative diseases remains to be explored.

The mechanism whereby compromised ECM remodeling disrupts CSF drainage remains unknown. It is possible that a buildup of dural ECM components in the CLL-treated group along MLVs could act as a physical molecular sieve that restricts fluid dynamics. Additionally, changes to ECM remodeling can also modify cellular pathways. For instance, ECM remodelers are involved in releasing growth factors, as MMP-2 and MMP-9 can proteolytically cleave ECM-bound transforming growth factor-β into its active form^31^; transforming growth factor-β mediates lymphangiogenesis through induction of *Vegfc* expression.^32^ Pharmacologically blocking MMP-2 and MMP-9 activity decreases lymphangiogenesis and vessel branching.^33^ Although no changes were observed in the length of MLVs after aCSF1R treatment, the structure of MLVs appeared smooth, with low LYVE-1 expression in some areas (Figure 5A). How these changes impact toxic protein removal or cell migration remains to be explored. Accordingly, further investigation into the signaling pathways associated with changes in ECM composition is required to elucidate the mechanism behind the modulation of CSF drainage and toxic protein clearance.

Monocytes can contribute to the dural macrophage population.^20^ This study confirmed that MLV recovery occurs 14 days after CLL treatment and that MLV recovery follows the replenishment of dural macrophage populations from bone marrow reservoirs. This was evidenced by the findings using the LysM-GFP reporter mice, showing monocyte-derived macrophages in the dura mater at day 14 after CLL treatment (Figure 3B). At day 7 after CLL treatment, the pattern of LYVE-1^+^ cell distribution varied in different areas (Figure 2B). Although there was a clear reduction of LYVE-1^+^ macrophages (CCL21^–^) and MLVs (CCL21^+^) in some areas, large disorganized LYVE-1^+^CCL21^–^ clusters, presumably LYVE-1^+^ macrophages, were seen in the hotspot areas (Supplemental Figure S3). Because dural sinus macrophage depletion is associated with monocyte infiltration as early as 3 days after CLL treatment, and these monocytes can differentiate into both LYVE-1^+^ and LYVE-1^–^ macrophages, these LYVE-1^+^ CCL21^–^ cell clusters were possibly derived from uneven monocyte infiltration and differentiation to macrophages at day 7 after CLL treatment.

Ossified vascular channels within the skull allow a direct route for bone marrow–derived myeloid cells to egress from bone marrow to the dura membrane.^34^, ^35^, ^36^ This direct route enables colony stimulation factor 1, produced by injury or infection, to reach calvarial bone marrow reservoirs to modify the immune environment, prompting the trafficking of bone marrow–derived myeloid cells to the dural mater.^37^^,^^38^ As such, we theorize that depletion of macrophages via CLL injection can initiate the arrival of bone marrow–derived myeloid cells to the dura mater, as implied by the increased proportion of monocytes in the dura mater 3 and 7 days after CLL treatment. This response to CLL treatment could foster the replenishment of dural macrophages and subsequent recovery of MLVs 14 days after CLL treatment. Overall, the differentiation of recruited bone marrow–derived myeloid cells could be a potential mean to facilitate the recovery of depleted dural macrophage populations after CLL treatment.

Notwithstanding the steady replacement of dural macrophages through local bone marrow–derived myeloid cells,^19^ self-renewing tissue-resident macrophages originating from yolk sac also exist in the dura mater.^39^ Intriguingly, recent evidence has identified these self-renewing subsets of macrophages by their expression of LYVE-1.^40^^,^^41^ Despite this, the study showed that monocytes can differentiate into both GFP^+^LYVE-1^+^ and GFP^+^LYVE-1^−^ macrophages, as seen in the LysM-GFP reporter mice (Figure 3B). However, the possibility that some dural macrophages may self-replicate after CLL-dependent depletion cannot be excluded. On the other hand, considering the comprehensive depletion of leptomeningeal macrophages by CLL treatment and the blood-brain barrier, which may hinder monocyte entry into the leptomeninges, it is reasonable to expect that leptomeningeal macrophages take longer to be restored compared with dural macrophages.

To summarize, this study demonstrated that i.c.m. administration of CLL preferentially depleted sinus-associated dural macrophages and leptomeningeal macrophages, resulting in reductions in MLV vasculature and CSF drainage. In contrast, i.p. injection of aCSF1R selectively depleted dural macrophages without impacting leptomeningeal macrophages, MLV structure, or CSF drainage. These results underscore the distinct roles of leptomeningeal and dural macrophages in regulating CSF drainage.

### Limitations of the Study

This study raises further questions about the regulation of MLVs and CSF drainage in adult mice. The original hypothesis proposed that dural macrophages in proximity to MLVs may provide lymphangiogenic or other regulatory factors necessary for the maintenance of MLVs in adult mice. However, i.p. injection of aCSF1R antibody, which effectively depleted dural macrophages, did not affect MLV structure or CSF drainage. Thus, the results did not support this hypothesis. Alternatively, it is well established that impaired CSF efflux to the dura mater can affect both the development of MLVs and maintenance of MLVs in aged mice.^12^ Given that 25 μL CLL treatment impairs CSF drainage (Figure 2), it is plausible that reduced drainage could lead to a decline in MLVs. However, treatment with 5 μL of CLL reduces leptomeningeal macrophages and CSF drainage,^17^ but does not significantly impact the structure of MLVs.^17^ Results in this study also showed intact MLVs with 5 μL of CLL (Supplemental Figure S6). Thus, the reduction in CSF drainage caused by CLL treatment is unlikely to result in the disruption of MLVs within the 7-day treatment window of this study. However, the possibility that 25 μL CLL treatment caused a more severe interruption of CSF drainage compared to the 5 μL CLL treatment, potentially leading to MLV reduction, cannot be excluded. Whether a more chronic interruption of CSF drainage or a longer duration of dural macrophage depletion may impact MLVs remains to be investigated. Alternatively, changes in the macrophage populations, gene expression profiles, or activity under diseased conditions or aging, rather than the mere absence of macrophages in the dura mater, may play a more significant role in regulating CSF drainage and the structure of MLVs.

At present, the mechanism by which 25 μL but not 5 μL i.c.m. CLL treatment impairs MLV structure remains unclear. Because treatment with 25 μL of PBSL did not alter the structure of MLVs in adult mice (Figure 2 and Supplemental Figure S9A), nor did this volume affect the body weight of the mice (Supplemental Figure S9B), the reduction in MLVs is unlikely to result from nonspecific neurologic damage caused by the large volume of injection. It is possible that, compared with the 5 μL of CLL treatment, the high dose of CLL treatment (25 μL) might enable lymphatic endothelial cells to phagocytose more CLL, leading to apoptosis. However, there is currently no direct evidence supporting this mechanism. Future studies investigating the phagocytic capacity of lymphatic endothelial cells for liposomes, as well as their viability following CLL injection, may provide further insights. Additionally, higher dose of CLL treatment may induce more severe alterations in CSF drainage, macrophage populations, and other cell types within both the CNS and dura mater. Comprehensive profiling of cell populations and gene expressions will be necessary to elucidate the underlying mechanism.

## Disclosure Statement

None declared.

## Supplemental Data

Supplemental Figure S1 Immune cell components in the dura mater of day 3 (**A**), 7 (**B**), and 14 (**C**) post–clodronate liposome (CLL)– and phosphate-buffered saline liposome (PBSL)–treated mice as determined by flow cytometry. Data are represented as means ± SEM (**A**–**C**). ∗*P* < 0.05, ∗∗*P* < 0.01 (two-tailed *U*-test). ns, no significance.

Supplemental Figure S2 Whole-mount staining of the transverse sinus shows a reduction in LYVE-1^+^ macrophages (positive for CD206 or F4/80) at day 3 after clodronate liposome (CLL) treatment compared with phosphate-buffered saline liposome (PBSL) controls in wild-type mice. Scale bars = 50 μm.

Supplemental Figure S3 Whole-mount staining of the transverse sinus shows the distribution of LYVE-1^+^ chemokine (C-C motif) ligand 21^+^ MLVs and LYVE-1^+^ macrophages at day 7 after clodronate liposome (CLL) treatment compared with phosphate-buffered saline liposome (PBSL) controls in wild-type mice. Scale bars = 250 μm. COS, confluence of sinus; TS, transverse sinus.

Supplemental Figure S4 Transcranial signal of fluorescein isothiocyanate–dextran distribution at the end point of intravital time-lapse imaging in wild-type mice. Scale bar = 2 mm.

Supplemental Figure S5 luorescein isothiocyanate (FITC)–dextran [2 MDa distribution in the α-smooth muscle actin (αSMA)–DsRed smooth muscle reporter mice at 2 hours after i.c.m. injection]. **A:** Brain. **B:** Brain cross-section. **C:** Leptomeninges. **D:** Dura mater in the inner side of the skull. **E: White arrows** represent the deep cervical lymph nodes. Scale bars: 2 mm (**A** and **E**); 1 mm (**B**); 500 μm (**C** and **D**). COS, confluence of sinus; SSS, superior sagittal sinus; TS, transverse sinus.

Supplemental Figure S6 Leptomeningeal macrophage and MLVs after 5 μL of clodronate liposome (CLL) i.c.m. injection. **A:** Leptomeningeal macrophages. Two-tailed *U*-test was used. **B:** MLVs and dural macrophages. Kruskal-Wallis test was used. Data are means ± SEM (**A** and **B**). ∗*P* < 0.05, ∗∗*P* < 0.01. Scale bars: 100 μm (**A**); 250 μm (**B**). COS, confluence of sinus; HS, hotspot; ns, no significance; PBSL, phosphate-buffered saline liposome; ROI, region of interest.

Supplemental Figure S7 Fold change of vascular endothelial growth factor C (VEGF-C) and matrix metalloproteinase-9 (MMP-9) transcripts for day 7 clodronate liposome (CLL)–treated mice compared with phosphate-buffered saline liposome (PBSL) controls. Data are represented as means ± SEM. ∗*P* < 0.05 (two-tailed *U*-test). Col, collagen; ns, no significance.

Supplemental Figure S8 Fluorescein isothiocyanate (FITC)–dextran and AL647-OVA retention in the leptomeninges (**A**) and dura mater (**B**) after anti–colony-stimulating factor 1 receptor (aCSF1R) treatment. Two-tailed *U*-test was used. Data are represented as means ± SEM (**A** and **B**). Scale bars: 100 μm (**A** and **B**, **bottom panels**); 250 μm (**B**, **top panels**). COS, confluence of sinus; ns, no significance.

Supplemental Figure S9 Phosphate-buffered saline liposome (PBSL; 25 μL) treatment did not impact MLVs or body weight of mice. **A:** MLVs in adult wild-type (WT) mice without any treatment. **B:** No changes in mouse body weight after i.c.m. injection of 25 μL PBSL or clodronate liposome (CLL). Scale bar = 250 μm (**A**). ns, no significance; ROI, region of interest.

Supplemental Video S2 Timelapse imaging showing FITC-dextran distribution in mice at day 7 post-CLL treatment.

Supplemental Video S1 Timelapse imaging showing FITC-dextran distribution in mice at day 7 post-PBSL treatment.
