# Supplementary figures and images for: Distinct Role of Dural and Leptomeningeal Macrophages in Maintaining Cerebrospinal Fluid Drainage to Meningeal Lymphatic Vessels
Source: Am J Pathol. 2025 Jun 18;195(9):1660–75. doi: 10.1016/j.ajpath.2025.05.017 (PMC12489351; doi:10.1016/j.ajpath.2025.05.017)

Supplemental Figure 1

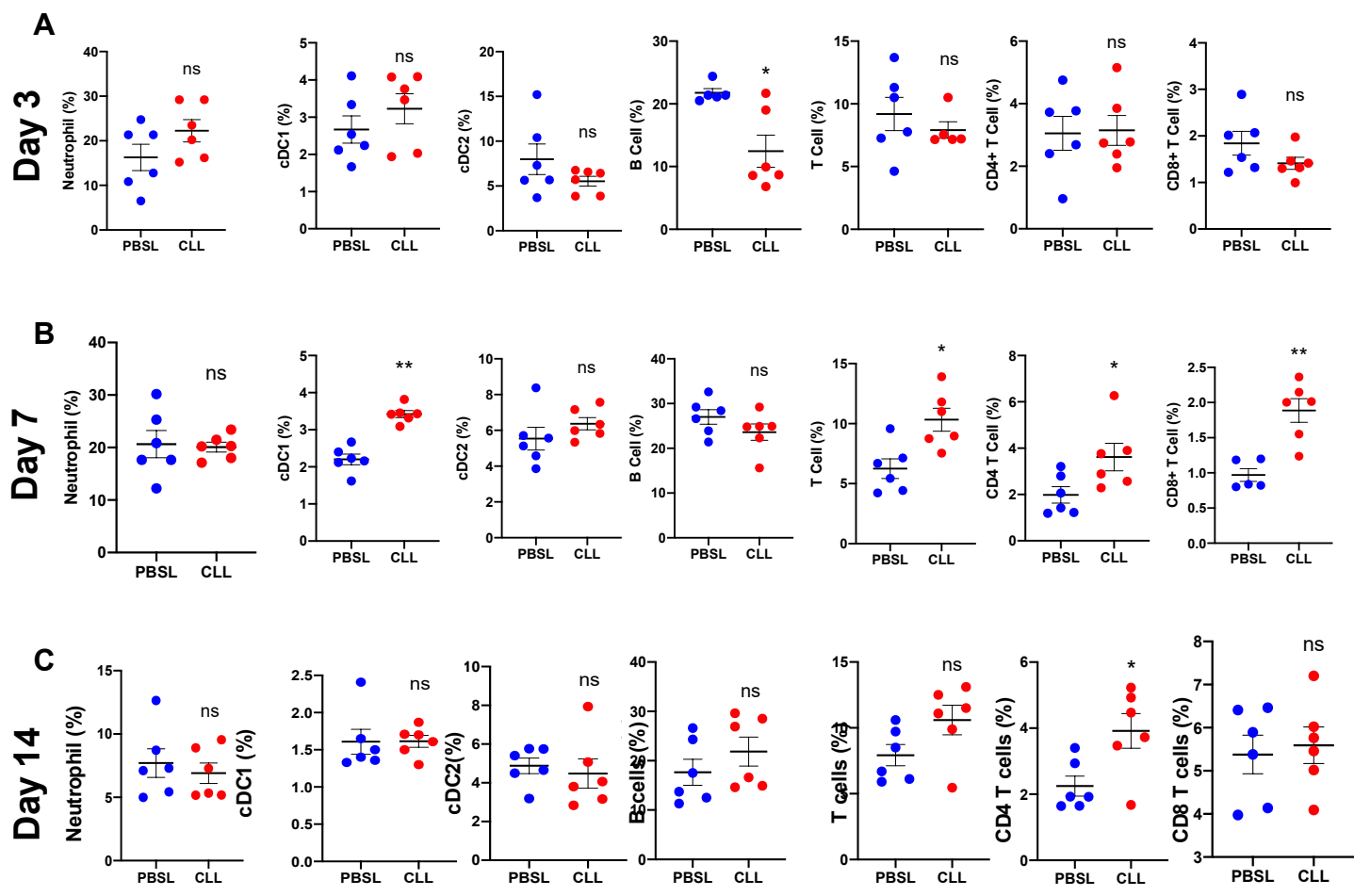

Supplement: Supplemental Figure S1 — Immune cell components in the dura mater of day 3 (A), 7 (B), and 14 (C) post–clodronate liposome (CLL)– and phosphate-buffered saline liposome (PBSL)–treated mice as determined by flow cytometry. Data are represented as means ± SEM (A–C). ∗P < 0.05, ∗∗P < 0.01 (two-tailed U-test). ns, no significance. [file mmc1.pdf]

Supplemental Figure 2

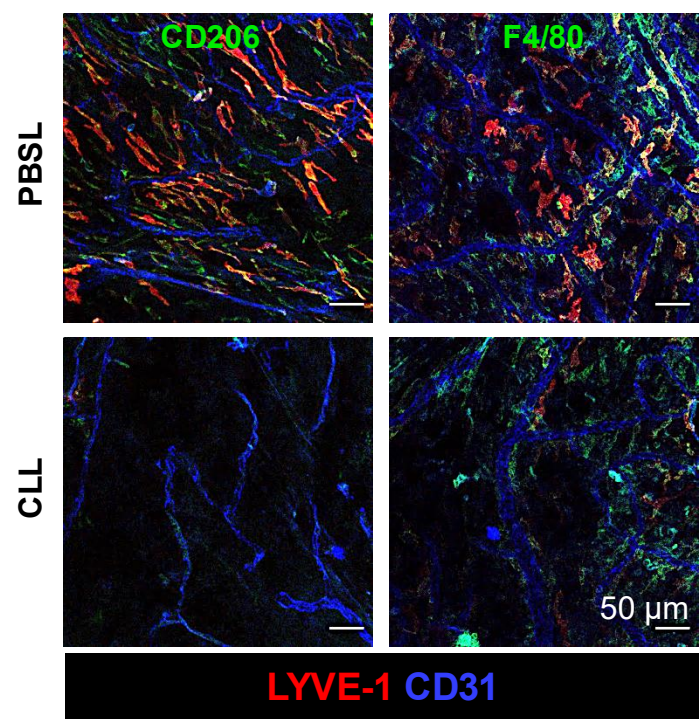

Supplement: Supplemental Figure S2 — Whole-mount staining of the transverse sinus shows a reduction in LYVE-1+ macrophages (positive for CD206 or F4/80) at day 3 after clodronate liposome (CLL) treatment compared with phosphate-buffered saline liposome (PBSL) controls in wild-type mice. Scale bars = 50 μm. [file mmc2.pdf]

Supplemental Figure 3

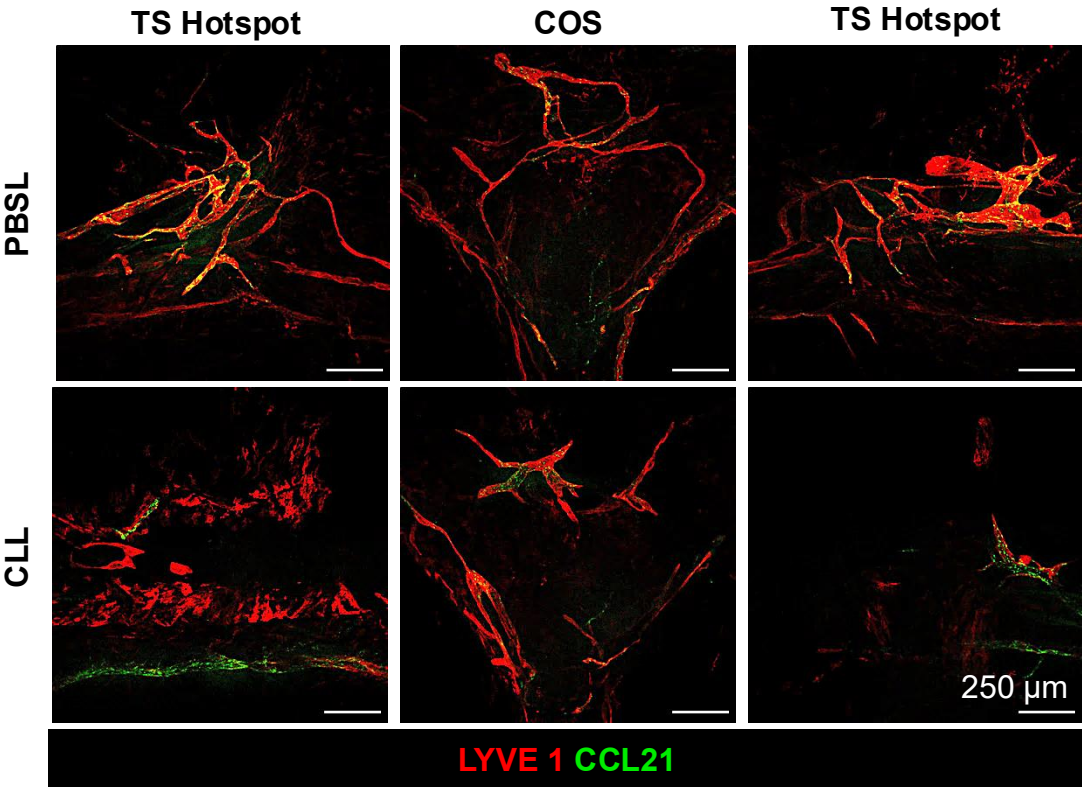

Supplement: Supplemental Figure S3 — Whole-mount staining of the transverse sinus shows the distribution of LYVE-1+ chemokine (C-C motif) ligand 21+ MLVs and LYVE-1+ macrophages at day 7 after clodronate liposome (CLL) treatment compared with phosphate-buffered saline liposome (PBSL) controls in wild-type mice. Scale bars = 250 μm. COS, confluence of sinus; TS, transverse sinus. [file mmc3.pdf]

Supplemental Figure 4

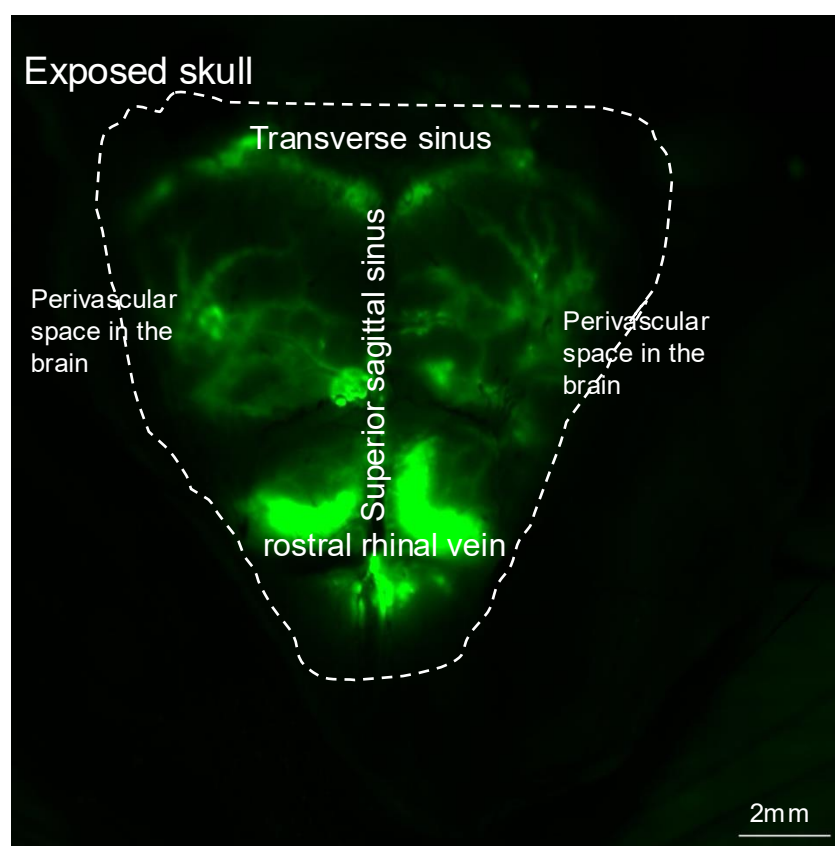

Supplement: Supplemental Figure S4 — Transcranial signal of fluorescein isothiocyanate–dextran distribution at the end point of intravital time-lapse imaging in wild-type mice. Scale bar = 2 mm. [file mmc4.pdf]

Supplemental Figure5

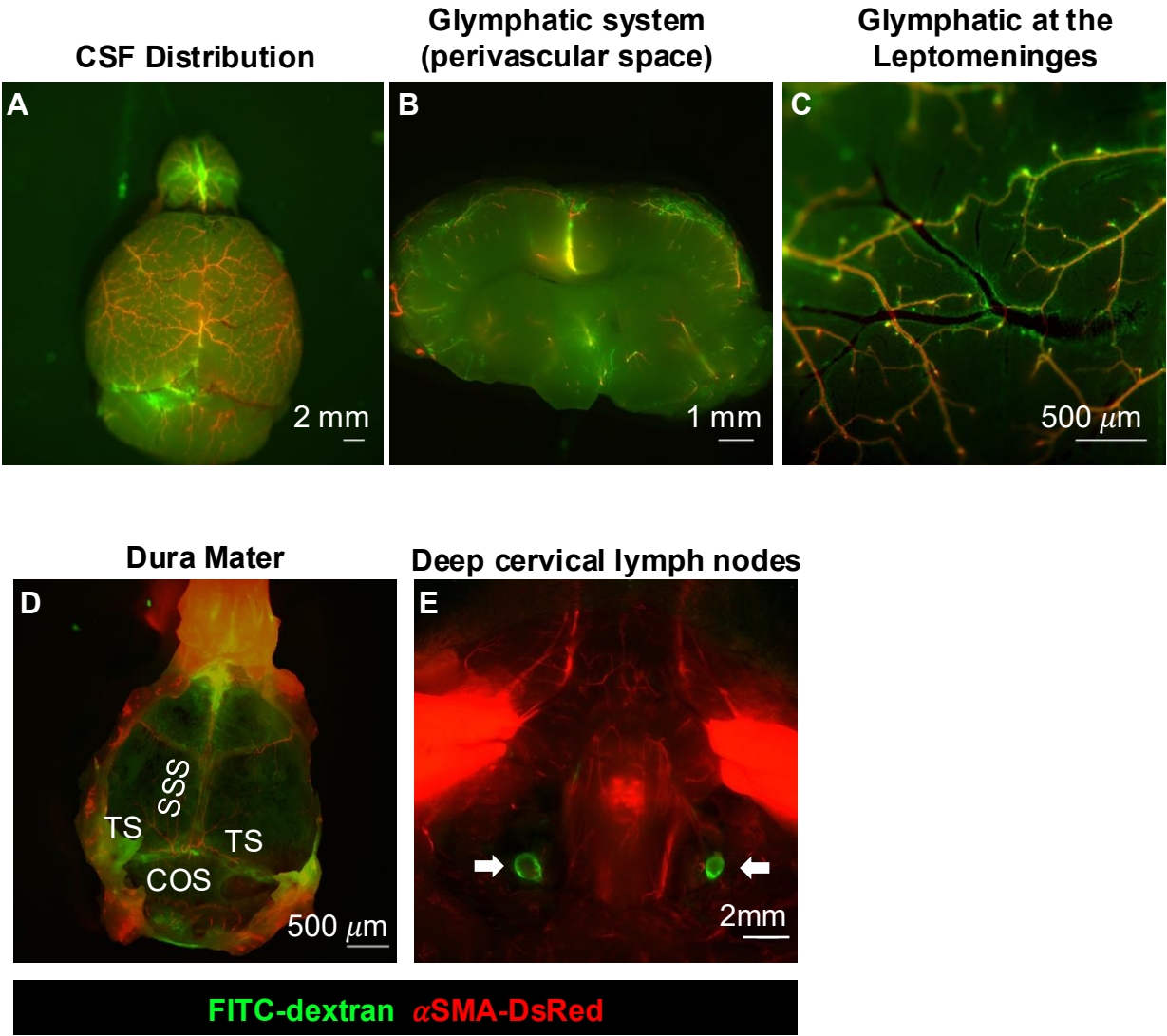

Supplement: Supplemental Figure S5 — luorescein isothiocyanate (FITC)–dextran [2 MDa distribution in the α-smooth muscle actin (αSMA)–DsRed smooth muscle reporter mice at 2 hours after i.c.m. injection]. A: Brain. B: Brain cross-section. C: Leptomeninges. D: Dura mater in the inner side of the skull. E: White arrows represent the deep cervical lymph nodes. Scale bars: 2 mm (A and E); 1 mm (B); 500 μm (C and D). COS, confluence of sinus; SSS, superior sagittal sinus; TS, transverse sinus. [file mmc5.pdf]

Supplemental Figure 6

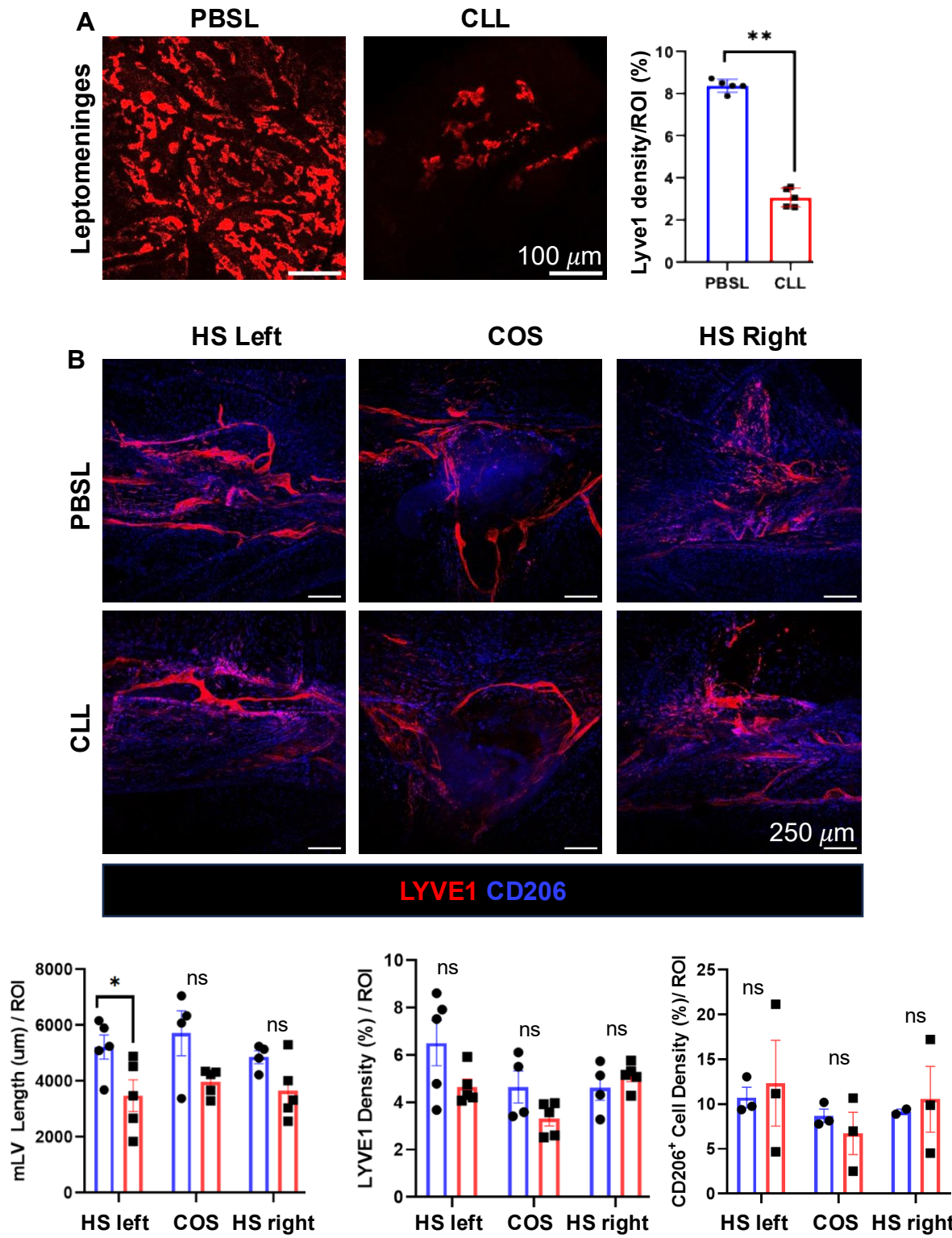

Supplement: Supplemental Figure S6 — Leptomeningeal macrophage and MLVs after 5 μL of clodronate liposome (CLL) i.c.m. injection. A: Leptomeningeal macrophages. Two-tailed U-test was used. B: MLVs and dural macrophages. Kruskal-Wallis test was used. Data are means ± SEM (A and B). ∗P < 0.05, ∗∗P < 0.01. Scale bars: 100 μm (A); 250 μm (B). COS, confluence of sinus; HS, hotspot; ns, no significance; PBSL, phosphate-buffered saline liposome; ROI, region of interest. [file mmc6.pdf]

Supplemental Figure 7

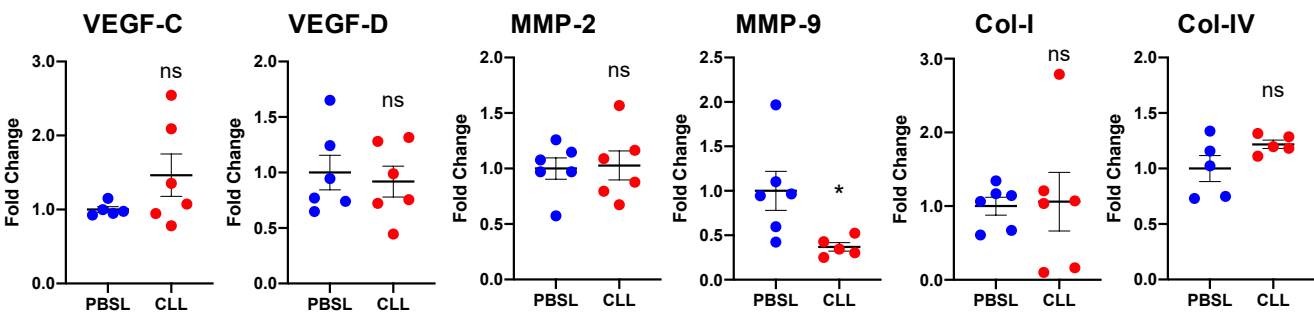

Supplement: Supplemental Figure S7 — Fold change of vascular endothelial growth factor C (VEGF-C) and matrix metalloproteinase-9 (MMP-9) transcripts for day 7 clodronate liposome (CLL)–treated mice compared with phosphate-buffered saline liposome (PBSL) controls. Data are represented as means ± SEM. ∗P < 0.05 (two-tailed U-test). Col, collagen; ns, no significance. [file mmc7.pdf]

Supplemental Figure 8

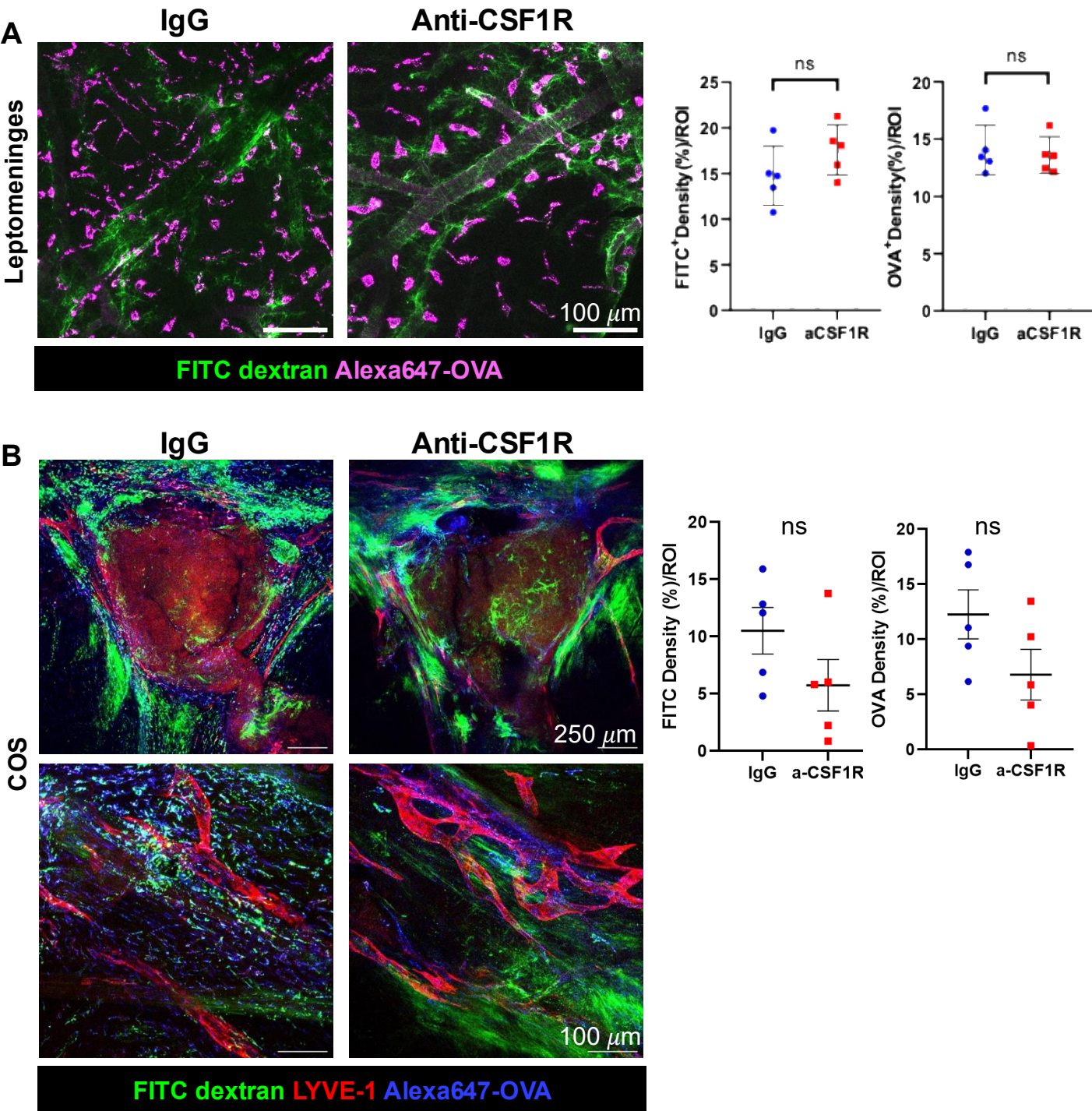

Supplement: Supplemental Figure S8 — Fluorescein isothiocyanate (FITC)–dextran and AL647-OVA retention in the leptomeninges (A) and dura mater (B) after anti–colony-stimulating factor 1 receptor (aCSF1R) treatment. Two-tailed U-test was used. Data are represented as means ± SEM (A and B). Scale bars: 100 μm (A and B, bottom panels); 250 μm (B, top panels). COS, confluence of sinus; ns, no significance. [file mmc8.pdf]

Supplemental Figure 9

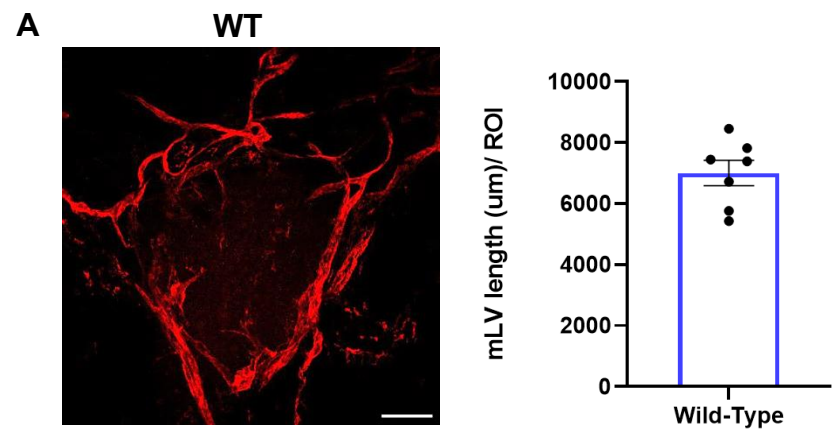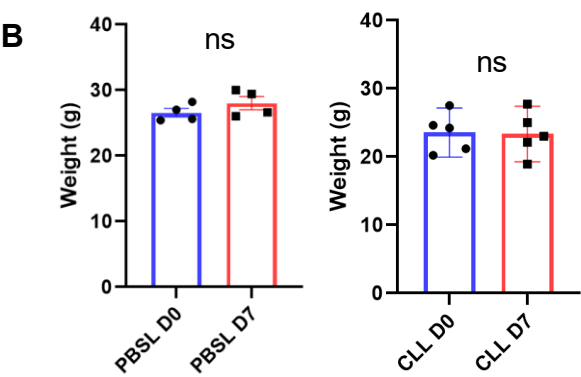

Supplement: Supplemental Figure S9 — Phosphate-buffered saline liposome (PBSL; 25 μL) treatment did not impact MLVs or body weight of mice. A: MLVs in adult wild-type (WT) mice without any treatment. B: No changes in mouse body weight after i.c.m. injection of 25 μL PBSL or clodronate liposome (CLL). Scale bar = 250 μm (A). ns, no significance; ROI, region of interest. [file mmc9.pdf]
